# Supplementary material for: Transdermal neuromodulation of noradrenergic activity suppresses psychophysiological and biochemical stress responses in humans
Source: Sci Rep. 2015 Sep 10;5:13865. doi: 10.1038/srep13865 (PMC4564766; doi:10.1038/srep13865)
Supplement: Supplementary Information [file srep13865-s1.doc]

**SUPPLEMENTARY INFORMATION**

**Transdermal neuromodulation of noradrenergic activity suppresses psychophysiological and biochemical stress responses in humans.**

William J. Tyler^*,#^, Alyssa M. Boasso^*^, Hailey M. Mortimore, Rhonda S. Silva, Jonathan D. Charlesworth,

Michelle A. Marlin, Kirsten Aebersold, Linh Aven, Daniel Z. Wetmore, and Sumon K. Pal

Thync, Inc.

Boston, MA USA 02199

**SUPPLEMENTARY INFORMATION**


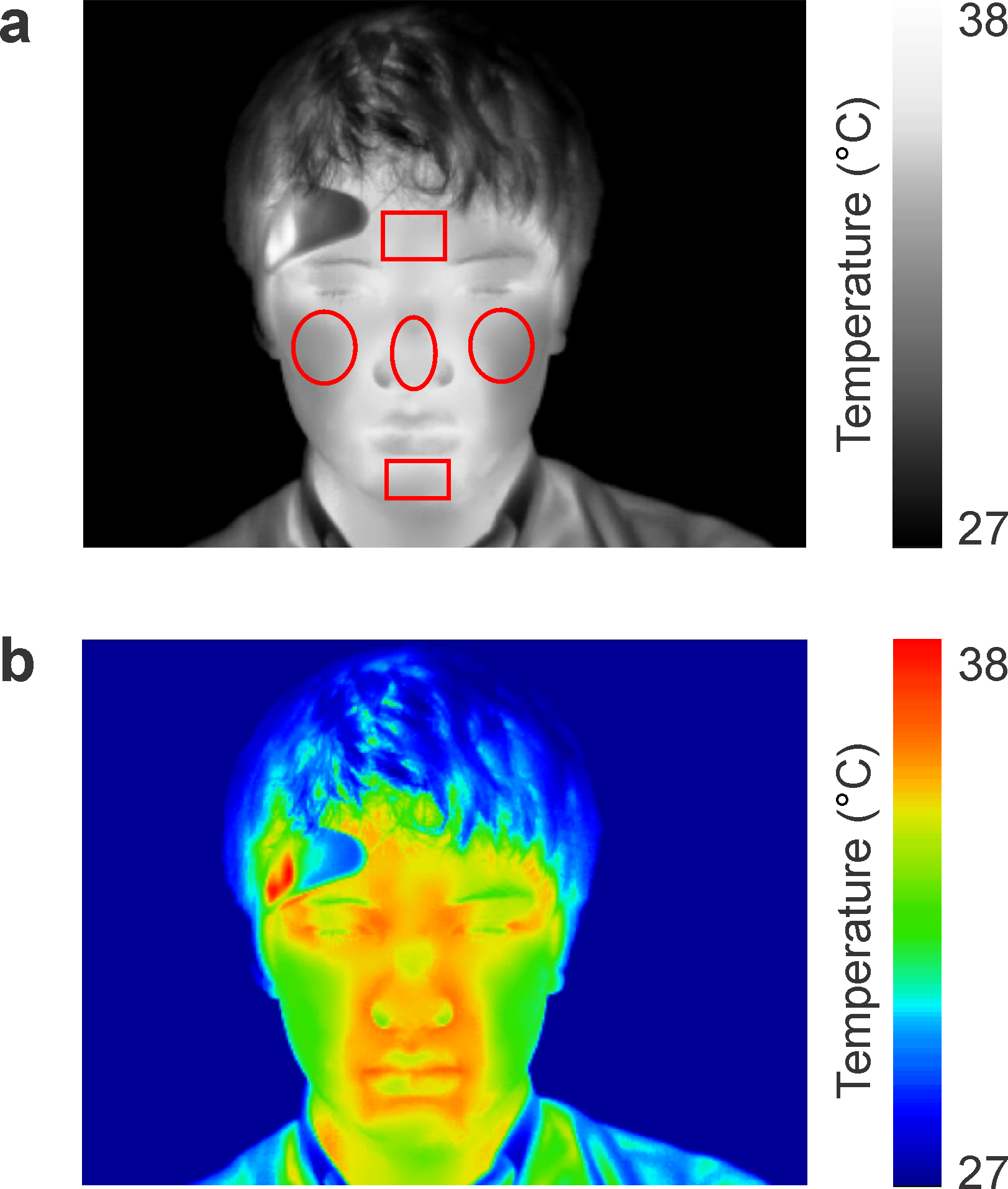


**Supplementary Figure 1. Approach to analysis of functional infrared thermography data. a**, The greyscale image of radiographic thermal data shows regions of interest (red) on the forehead, nose, cheeks, and chin that were used to measure the skin temperature at different time points from the time-lapsed data acquired. **b**, The same image is shown as above with a pseudo-color look-up table applied to make the visualization of temperature differences easier for readers such that *blue* represents 27**°** C, *green* represents 33**°** C, and *red* represents 38**°** C.

**Supplementary Video Legends**

**Supplementary Video 1**

The video shows time lapsed data obtained using functional infrared thermography of the face of a representative subject in the sham treatment group. The video is shown at approximately 40X real time speed. At the beginning of the video the baseline period is shown. During the baseline period the device is actively powered in a listening mode waiting for the Bluetooth signal and a thermal signature can be observed emitted from the device due to power consumption. The appropriate sham waveform is then streamed to the device over a Bluetooth network during the stimulation period. The sham treatment begins when the *white square* appears in the upper right hand corner of the video and when the *red square* appears the sham stimulation is terminated. The device is remotely powered off at the end of the treatment period and the thermal signature from the device can be observed dissipating. The radiometric data are shown with a pseudo-color look-up table applied that is the same as shown in Figure 3 where *blue* represents 27**°** C, *green* represents 33**°** C, and *red* represents 38**°** C.

**Supplementary Video 2**

The video shows time lapsed data obtained using functional infrared thermography of the face of a representative subject in the TEN treatment group. The video is shown at approximately 40X real time speed. At the beginning of the video the baseline period is shown. The TEN treatment begins when the *white square* appears in the upper right hand corner of the video and when the *red square* appears the TEN stimulation is terminated. During the baseline period the device is actively powered in a listening mode waiting for the Bluetooth signal and a thermal signature can be observed emitted from the device due to power consumption. The appropriate TEN waveform is then streamed to the device over a Bluetooth network during the stimulation period. The device is remotely powered off at the end of the treatment period and the thermal signature from the device can be observed dissipating. The radiometric data are shown with a pseudo-color look-up table applied that is the same as shown in Figure 3 where *blue* represents 27**°** C, *green* represents 33**°** C, and *red* represents 38**°** C.
